# The space between us: The effect of perceived threat on discomfort distance and perceived pleasantness of interpersonal vicarious touch

**DOI:** 10.1016/j.heliyon.2024.e36487

**Published:** 2024-08-16

**Authors:** Yasemin Abra, Laura Mirams, Merle T. Fairhurst

**Affiliations:** a Centre for Tactile Internet with Human-in-the-Loop (CeTI), 6G life, Faculty of Electrical and Computer Engineering, Technische Universität Dresden, Dresden, Germany; b School of Natural Sciences and Psychology, Liverpool John Moores University, Liverpool, United Kingdom

**Keywords:** COVID-19, Affective touch, Threat, Discomfort distance, Interpersonal boundary

## Abstract

The space we keep between ourselves and others allows us either to engage in close shared experiences or to distance ourselves for safety. Focusing primarily on the latter, previous studies have identified a link between interpersonal boundaries and perceived threat, perceptual discrimination including pain perception as well as how we move and behave as a result. Although interpersonal distancing has been studied in a range of contexts, a mechanistic way of how such spatial behaviour might alter how we perceive affective touch has yet to be investigated. Here we probe the effect of perceived threat of COVID-19 on interpersonal boundary preferences and perceived pleasantness of vicarious affective touch. Our results demonstrate that increased perceived threat from COVID-19 is associated with larger boundaries of discomfort distance. Moreover, we show a positive association between perceived threat and pleasantness of vicarious touch coming from a member of the household, but no association with outsider touch. Importantly, rather than focusing on the purely “positive” and prosocial functions of affective touch, these results bolster a novel perspective that socially-relevant cues guide both approach and avoidance behaviours.

## 1 Introduction

The spaces surrounding our bodies determine how we interact with and perceive the world and others [1]. Adaptive and flexible in nature, they prepare our bodies for goal-directed actions, social interactions as well as for defensive responses against potentially harmful stimuli [2]. As such, highlighting the *nature* of the stimulus within bodily boundaries, the literature distinguishes between action and social spaces, as well as how they regulate one another [3]. Within this so-called social space that serves to maintain proper distancing between each other, smaller interpersonal boundaries allow us to engage in close shared experiences while larger boundaries allow us to distance ourselves for safety. Previous research has focused almost exclusively on the latter, identifying a link between personal space (i.e. the space in which a social interaction is felt to be uncomfortable) and perceived threat, perceptual discrimination including pain perception as well as how we move and behave [4]. As an example of these avoidance behaviours, there were marked differences in the distance we kept between ourselves and others during the COVID-19 pandemic [5]. These interpersonal behaviours are expected to vary not only due to cultural or regulated norms, but also due to changing individual preferences. Importantly, we assume that interpersonal space will influence close interpersonal interactions such as social touch. Here we investigate the flexible and nuanced nature of discomfort and reaching distances (henceforth DD and RD, respectively) and how variation in these distances are associated with perceived and actual threat from COVID-19 as well as how this association relates to the perceived pleasantness of vicarious affective touch.

### 1.1 The distance between us and how we measure it

As we continuously adapt our behaviour to fit the environment, the boundaries surrounding our bodies need to be plastic and capable of dynamically adjusting to the altering sensorimotor and social context [6]. To capture this, previous studies of interpersonal boundaries have had participants imagine themselves to be the figure in the centre of a circle and to mark the distance where they would start feeling uncomfortable if another figure was approaching them [7]. Alternatively, paper-and-pencil questionnaires with two human figures have been presented with respondents asked to imagine that they were one of the agents and to rate how close the other person could approach them, in order for them to still feel comfortable [8]. Several studies have investigated interpersonal boundary preferences during the COVID-19 pandemic; a global event during which, to avoid transmission of the virus, regulated and social norms limited close personal interaction. In these studies, boundary preferences have always been quantified using visual scales. Welsch and colleagues surveyed 136 German participants provided ratings on a graphic sliding scale and instructed to indicate preferred DD before and during the pandemic, as well as how they anticipated feeling after the pandemic [9]. The results showed a rapid adaptation to distancing rules and an enlargement of DD that might potentially linger after the pandemic. Other findings along the line of adaptation to current norms include a study by Cartaud et al. where participants assigned reduced appropriateness to the social distance of avatars with masks compared to those without [10]. Hromatko and colleagues measured preferred interpersonal boundaries using a graphical task where participants were asked to specify the distance they would feel comfortable having different individuals approaching them [11]. Identifying differences from pre-COVID measurements [8], the study further highlights how the space between us varies as a function of the type of interaction distinguishing between social, personal and intimate distance. Beyond these contextual factors, there are clearly a number of additional triggers that result in the measured changes in interpersonal boundaries ranging from increased health anxiety [12] to social distancing rules to prevent transmission [13] highlighting that we respond defensively to both perceived and objective levels of threat.

### 1.2 Threat and interpersonal boundaries

Given the protective function of interpersonal boundaries to avoid potentially harmful stimuli (Geers & Coello, 2023), measured differences in DD during the pandemic have been suggested to vary as a function of perceived threat from COVID-19 [11]. Iachini and colleagues reported that DD regulation was affected by how people subjectively perceived COVID-19 risk and the related level of anxiety, but not by actual threat [14]. Similarly, Makhanova and Shepherd report that American participants with higher perceived COVID-19 threat show higher pathogen avoidance operationalized as perceived vulnerability [15]. Beyond changes in DD, reaching distance (RD) or the space in which we voluntarily engage with our environment for goal-oriented actions, is expected to decrease as a function of perceived threat and anxiety [4]. Specific to the context of the pandemic, changing touch attitudes toward the handling of objects is reinforced by suggestions to avoid contact with contaminated surfaces [16].

This ability to detect threats is critical for survival [17] but what makes a stimulus within interpersonal boundaries threatening? Unlike defining actual threat based on objective parameters, characterising threats based on a specific combination of perceptual features is not possible, partly because the *perception* of threat depends on how one relates to the context of the threat [1], highlighting the affective content of threat perception. Therefore another potential consequence of perceived threat from COVID-19 is a change in which stimuli are classed as potentially harmful, and so elicit a defensive reaction when they occur within the defensive peripersonal space [18,19]. In the context of touch, this might manifest as a wariness of unfamiliar interpersonal touch [20,21] and a decrease in perceived pleasantness of potentially harmful touch events.

### 1.3 Subjective nature of affective touch

Affective touch is a powerful sensory experience that shapes trajectories of development [22], regulates pain and stress [23] and, by definition, requires close physical interaction. The growing body of research on the topic has identified both bottom-up and top-down features that mark interpersonal affective touch and resulting perceived pleasantness. In some of the earliest work in humans identifying a special class of C tactile (CT) afferent that are selectively activated in response to gentle stroking, Löken and colleagues report optimal firing of these CTs fibres at moderate velocities ranging from 3 to 10 m/s [24]. More recent research focuses on detailing top-down factors that manipulate the perceived pleasantness of social touch, such as who is touching and our relationship to them [25]. Importantly, the subjective and selective nature of our experience of touch has also been shown for vicarious touch experiences [26] with tuned neural responses to videos of interpersonal touch at CT-optimal stroking speeds [27]. Expanding beyond the typical focus on its “positive” and prosocial functions, previous work has probed the highly subjective nature of affective touch revealing that behavioural and neural responses are strongly modified by an array of situational and contextual factors [28]. Moreover, the utility and hedonic experience of interpersonal touch has been shown to be modulated by the context and internal state of the individual [29,30]. Within the context of the COVID-19 pandemic, several studies have investigated contextual modulation showing that how touch is perceived is influenced by touch deprivation [21,31]. Moreover, Von Mohr and colleagues suggest that who is touching us and the kind of touch used results in graded differences in longing for touch comparing intimate, friendly and professional contexts. Based on the so-called “social salience hypothesis” which proposes a shift of the neurotransmitters oxytocin and dopamine to facilitate attention-orienting responses to external contextual social cues [32], this system may be also at work when socially-relevant information cue danger rather than trust, and thereby shift the balance from sensory seeking to sensory avoidance [33,34].

### 1.4 Present study

**Fig. 1:**
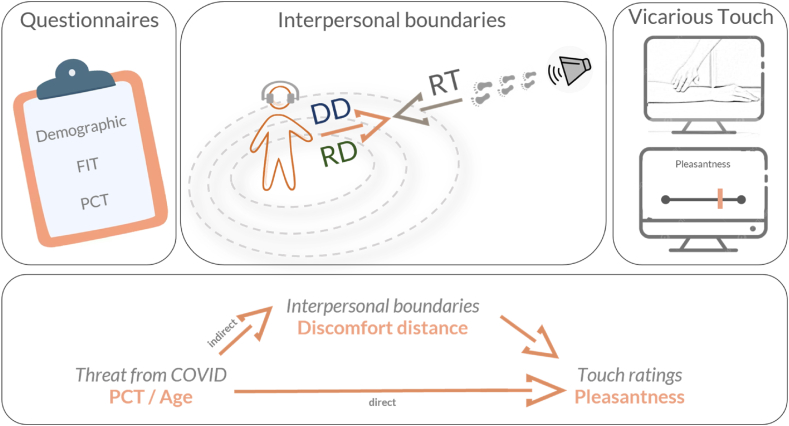
**Study design and hypothesis.** An online study consisting of (left) a questionnaire section with basic demographic information including age, Fear of Interpersonal Touch (FIT) and Perceived Coronavirus Threat (PCT); (middle) an auditory interpersonal boundary task from which Discomfort distance (DD) and reaching distance (RD) could be inferred as a function of response time (RT) and finally (right) a vicarious touch task in which videos were presented and rated in terms of pleasantness, comfort and likeability. (Bottom) We investigate either a direct or indirect relationship between perceived and actual threat from COVID-19 and how we perceive affective touch as a function of preferred interpersonal distance.

In an online study, we therefore investigate the effect of threat on DD and RD, as well as perceived pleasantness of vicarious affective touch. Our study further contributes to the literature by modulating interpersonal boundaries with the use of auditory triggers, specifically the footsteps of an approaching person, shown to be superior to vision in a variety of practical cases [35]. We test the conceptual framework proposed by Ianetti and colleagues suggesting that the same object present in our environment may variably be perceived as threatening or not [18,19]. We hypothesise that participants with higher perceived threat from COVID-19 will show larger preferred boundaries of DD and smaller boundaries of RD as proxied by faster and slower response times, respectively, and be more likely to consider affective touch within their personal space as potentially harmful, resulting in higher fear of interpersonal touch and reduced ratings of pleasantness of vicarious affective touch. Similarly, we expect that actual threat from COVID-19 operationalized as age, where older participants are objectively at a higher risk of contracting and suffering from COVID-19, will predict larger boundaries of DD and lower preference towards interpersonal touch [36]. We also look at perceived threat as it relates to touch perception, which especially within the specific context of COVID-19, inherently revolves around uncertainty on whether or not we can know if the individual touching us is contagious. Therefore, we predict that individuals in one's household present a lower threat than outsiders irrespective of familiarity [21], resulting in increased pleasantness of vicarious affective touch from a household member. We collected our data from two countries: while Germany implemented an immediate lockdown and kept the increase in number of deaths lower than predicted, the delayed lockdown in the UK resulted in a higher plateau (Balmford et al., 2020). Hence, by collecting data from these two countries experiencing and responding differently to the threat of COVID-19 (Knolle et al., 2021), we investigate how this interacts with subjective appraisal of threat. More specifically, we hypothesise that participants from both countries will report an increase in fear of interpersonal touch and larger boundaries of DD. However, considering the times of data collection for the two countries which coincided with the rapid spread of the Alpha variant in the UK and the gradual lifting lockdown measures in Germany, we expect that this increase would be more pronounced in UK responders as a function of perceived threat from COVID-19. Finally, creating a bridge between the relatively understudied field of tactile defensiveness [37] and social threat perception [38], we investigate the nature of the relationship between DD and perception of affective touch and the influence, if any, of fear of interpersonal touch (see Fig. 1).

## 2 Results

### 2.1 COVID-19 threat and fear of interpersonal touch

To investigate whether actual and perceived threat from COVID-19 was associated with the fear of interpersonal touch, we built a multiple linear regression model with the overall score for Fear of Interpersonal Touch Questionnaire (FIT) as the outcome variable and the scores for Perceived Coronavirus Threat Questionnaire (PCT) for perceived threat, age marking actual threat from COVID-19, country of residence and its interaction with perceived threat as predictor variables.

**Fig. 2:**
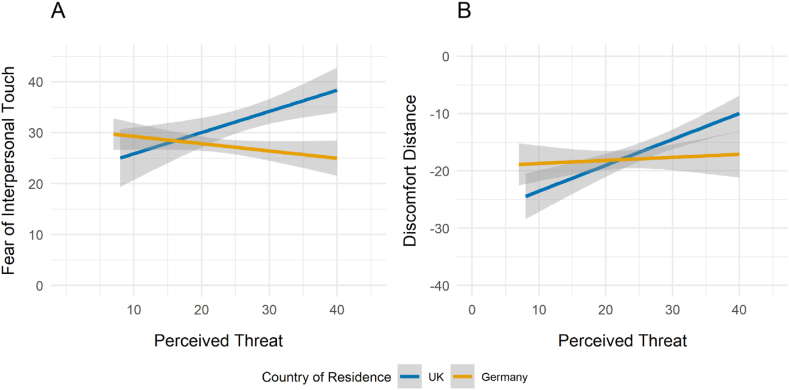
**Avoidance behaviours and the influence of perceived threat from COVID-19** on A) Fear of interpersonal touch and B) Discomfort Distance as a function of country of residence (for visual purposes, response times in seconds are reversed to indicate discomfort distance). Plots include regression lines with shaded 95 % confidence intervals.

The analysis revealed that the overall model was significant: F(4,91) = 7.32, *p* < .001, *R*^*2*^ = .21. The effect of perceived threat on fear of touch depended significantly on country of residence: with the UK sample perceived threat is associated with higher aversion to interpersonal touch whilst with the German sample, this effect is reversed (β = −.020, CI: .031 to −.009, *p* < .001) (Fig. 2A). The age effect was not significant (β = −.0017, CI: .005 – .002, *p* = .36). There was also a main effect of PCT (β = .014, CI: .006–.022, *p* < .001): the greater the perceived threat from COVID-19, the higher the fear of interpersonal touch, and of country (β = .29, CI: .005–.581 *p* = .047): being in Germany, compared to the UK, is associated with an increase in fear of interpersonal touch, holding other variables constant.

### 2.2 COVID-19 threat and changes in interpersonal boundaries

Multiple linear regression analysis with the scores for PCT for perceived coronavirus threat, age marking actual threat, country of residence and its interaction with perceived threat as predictor variables were applied to two models: 1) predicting the RT when participants were asked to indicate “Stop” when they felt uncomfortable with the distance between them and the approaching person (i.e. Discomfort Distance), and 2) predicting the RT when participants were asked to indicate “Stop” when they felt like they could touch the approaching person (i.e. Reaching Distance).

The first model was significant overall (F(4,91) = 6.4, *p* < .001, *R*^*2*^ = .19), with age (β = .007, CI: .002–.013 *p* = .01), PCT (β = −.028, CI: .041 to −.015, *p* < .001), and the interaction between perceived threat and country (β = .023, CI: .005–.040, *p* = .01) being significant variables: older age is associated with a smaller DD, and higher perceived threat from COVID-19 predicts greater DD overall. The effect of perceived threat on DD, however, is different for UK and German samples such that it is more pronounced in the UK sample compared to German (Fig. 2B). The second model (F(4,91) = 4.92, *p* = .001, *R*^*2*^ = .14) showed that older age (β = .009, CI: .003–.015, *p* = .002) is associated with a smaller RD, and a main effect of perceived threat from COVID-19 that is associated with greater RD (β = −.024, CI: .037 to −.011, *p* < .001). The effect of perceived threat on RD also depended significantly on country of residence: with the UK sample perceived threat is associated with a greater RD whilst with the German sample, this effect was reversed (β = .024, CI: .006–.042, *p* = .001) (Fig. S1).

### 2.3 Vicarious affective touch ratings as a function of stroking speed and context

**Fig. 3:**
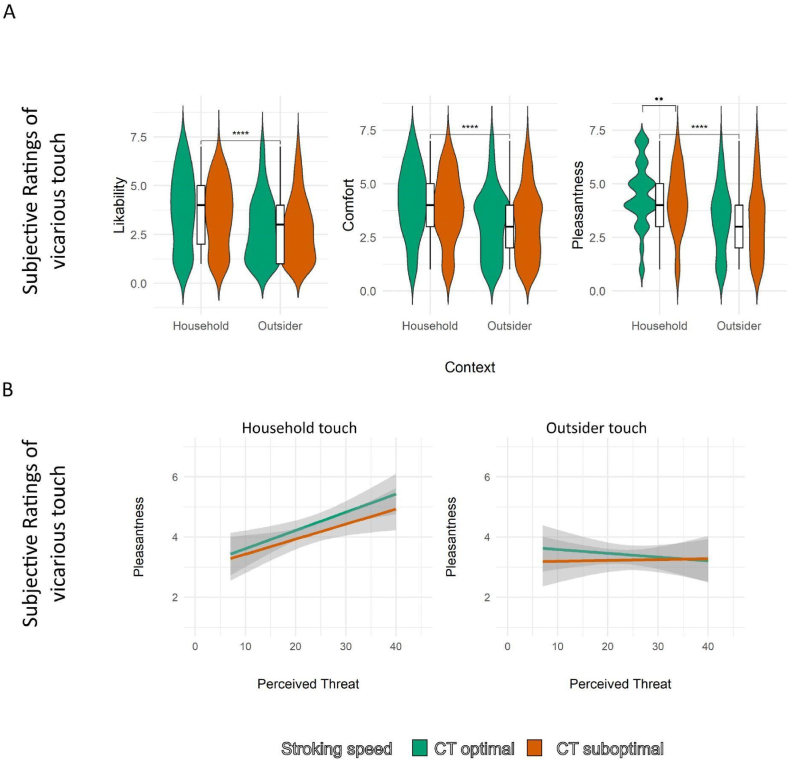
**Approach behaviours and ratings of vicarious affective touch as a function of A) stroking speed and context for likeability, comfort and pleasantness ratings and B) perceived COVID-19 threat** (n = 96). A) The violin plots show the distribution of likability, comfort and pleasantness ratings for Context (Household and Outsider), differentiated by Stroking speed (CT optimal and CT suboptimal). The overlaid box plots indicate medians and interquartile ranges. Asterisks indicate significance level with **** denoting *p* < .0001; ** denoting *p* < .01. B) Linear regression plots show the relationship between perceived threat from COVID-19 and pleasantness ratings for Context (Household and Outsider), differentiated by Stroking speed (CT optimal and CT suboptimal). The plots include regression lines with shaded 95 % confidence intervals.

In order to assess the effect of context and stroking speed on the three dimensions of vicarious touch ratings namely likability, comfort and pleasantness, linear mixed effect models with the ratings as dependent variables, the fixed effects Context (household, outsider) and Stroking Speed (CT-optimal vs. CT-suboptimal), and by-subject random intercepts were fitted using the lme4 package (v1.1-26; [39]). The models predicting likability, comfort and pleasantness of vicarious touch revealed a main effect of Context respectively (F(1,282) = 63.57, β = −.84, CI: 1.046 to −.630, *p* < .001; F(1,277) = 66.3, β = −.83, CI: 1.028 to −.628, *p* < .001; F(1,281) = 98.4, β = −.98, CI: 1.170 to −.783, *p* < .001, see Fig. 3A) indicating that household touch predicts better likability, comfort, and pleasantness. Pleasantness was also predicted by optimal-stroking speed touch (F(1,281) = 6.82, β = −.26, CI: .451 to −.064, *p* < .001). There was no effect of stroking speed on likability (β = −.1, CI: .304 – .112, *p* = .4) or comfort (β = −.14,CI: .340 – .059, *p* = .17).

### 2.4 Perceived threat and ratings of vicarious affective touch

In order to ascertain the effect of perceived threat from COVID-19 on the perception of vicarious *Household* and *Outsider* touch at *CT-optimal* and *suboptimal* stroking speeds, linear regression models were fitted with the vicarious touch ratings namely likability, comfort and pleasantness as outcome and Perceived COVID-19 Threat as the predictor variables. The analyses showed that while perceived threat from COVID-19 was associated with higher ratings for Household touch both at optimal and suboptimal stroking speeds, there was no association with Outsider touch at either stroking speed (see Fig. 3B). Specifically, higher perceived threat predicted better likability (F(1,91) = 11.87, β = .08, CI: .035–.129, *p* < .001, *R*^*2*^ = .11), comfort (F(1,91) = 9.16, β = .064, CI: .022–.106, *p* = .003, *R*^*2*^ = .08), and pleasantness (F(1,93) = 10.2, β = .06, CI: .023–.098, *p* = .002, *R*^*2*^ = .09) of CT-optimal Household touch, and better likability (F(1,92) = 5.53, β = .06, CI: .008–.095, *p* = .02, *R*^*2*^ = .05), comfort (F(1,90) = 6.9, β = .06, CI: .014–.100, *p* = .01, *R*^*2*^ = .06), and pleasantness (F(1,92) = 6.5, β = .05, CI: .011–.089, *p* = .013, *R*^*2*^ = .06) of CT-suboptimal Household touch (Fig. S2). Perceived threat from COVID-19 was not associated with Outsider touch of neither CT optimal (likability (F(1,94) = .11, β = .007, CI: .035 – .050, *p* = .73, *R*^*2*^ = −.01), comfort (F(1,92) = 0, β = .0001, CI: .041 – .041, *p* = .99, *R*^*2*^ = −.01), and pleasantness (F(1,94) = .37, β = −.01, CI: .053 – .028, *p* = .54, *R*^*2*^ = −.01)) or suboptimal stroking speed (likability (F(1,94) = .04, β = , CI: .037 – .046, *p* = .84, *R*^*2*^ = −.01), comfort (F(1,94) = .01, β = .002, CI: .040 – .044, *p* = .9, *R*^*2*^ = −.01), and pleasantness (F(1,92) = .02, β = .003, CI: .041 – .046, *p* = .9, *R*^*2*^ = −.01)).

### 2.5 COVID-19 threat, fear of touch, interpersonal and reaching distances on CT-optimal stroking speed household touch ratings

A multivariate linear regression model was built for the vicarious affective touch ratings (likability, comfort, and pleasantness) for CT-optimal touch from a household member as the outcome variables, and the scores for PCT marking perceived threat, age marking actual threat, country of residence and its interaction with PCT as well as the FIT score, response times marking DD and RD as predictor variables. 95 % confidence intervals for each coefficient in the multivariate linear model were calculated from bootstrap samples using the boot package (v1.3-27 [40];Canty & Ripley, 2021). The analysis revealed that the model is statistically significant for each outcome: *Likability* F(7,83) = 2.8, *p* = .01, *R*^*2*^ = .12; *Comfort* F(7,83) = 2.4, *p* = .026, *R*^*2*^ = .10), *Pleasantness* F(7,83) = 2.5, *p* = .038, *R*^*2*^ = .09), with smaller boundaries of DD predicting pleasantness (as proxied by response times; β = 7.9e-05, CI: .13 – .11, *p* = .03), a trend for larger boundaries of RD predicting comfort (as proxied by response times; β = −6.9e-05, CI: .038 – .05, *p* = .06), and a trend for perceived threat predicting likability (β = .08, CI: 1.13–6.77, *p* = .07) and pleasantness (β = .06, CI: 6.5e-06 – 1.65e-04, *p* = .07).

For exploratory purposes, we removed the interaction term between PCT and country of residence from the model and found that increased perceived threat predicted better likability (β = .07, CI: .88 – 4.49, *p* = .007), comfort (β = .06, CI: .83 – .87, *p* = .01) and pleasantness (β = .07, CI: 3.3e-05 – 1.4e-04, *p* < .001) of optimal stroking speed household touch, with longer response times, i.e. smaller boundaries of DD predicting better pleasantness (β = .08, CI: 1.4e-04 to −9.4e-06, *p* = .03).

### 2.6 Perceived threat, discomfort distance and pleasantness ratings of vicarious affective touch

**Fig. 4:**
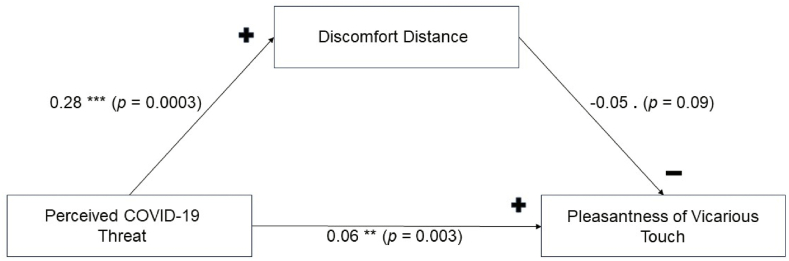
**The link between perceived COVID-19 threat, Discomfort Distance, and pleasantness of vicarious touch.** Mediation analysis showing that the effect of perceived threat on the pleasantness of vicarious touch is not mediated by DD. Asterisks indicate significance level with *** denoting *p* < .001; ** denoting *p* < .01; . denoting .1 > *p* > .05.

Mediation analysis was performed between perceived threat from COVID-19, response times for the discomfort distance task, and the pleasantness ratings for household touch at optimal stroking speed as independent, mediator, and dependent variables, respectively. As Fig. 4 illustrates, the regression coefficient between PCT and the perceived pleasantness of household vicarious touch of optimal stroking speed was significant, while the regression coefficient between DD and pleasantness of touch was only marginally significant. The indirect effect was (.006) x (−.05) = −.003. We also tested the significance of this indirect effect using bootstrapping procedures using the mediation R package [41]. The bootstrapped unstandardised indirect effect was −.003, and the 95 % confidence interval ranged from −.02 to .01. Thus, the indirect effect of perceived threat on the touch ratings via DD was non-significant (*p* = .70).

## 3 Discussion

The present study investigated the effect of perceived threat from COVID-19 on interpersonal distance preferences and perceived pleasantness of vicarious touch. We present an analysis of preferences for interpersonal distance over a sample from two countries, namely Germany and UK, and how it relates to actual and perceived threat, as well as vicarious affective touch perception. We show, on the one hand, that perceived threat from COVID-19 was predictive of larger DD and RD boundaries as well as fear of interpersonal touch but, on the other hand, also of increased ratings of pleasantness of household touch We also find that these effects interact with the country of residence. Distinguishing between perceived and actual threat, we show for the latter that participants report smaller boundaries of DD and RD. Importantly, rather than focusing on the purely “positive” and prosocial functions of affective touch, our results bolster a novel perspective that socially-relevant cues guide both approach and avoidance behaviours.

### 3.1 Perceived threat and changes in discomfort distance

Adding to the COVID-19 literature on interpersonal distances [20], we find that higher perceived threat from COVID-19 is associated with larger boundaries of DD [42]. Similarly, Vagnoni and colleagues found that threatening stimuli induced participants to misperceive the time-to-collision to their body with respect to non-threatening stimuli [43]. Indeed, increased threat perception in COVID-19, and beliefs about the infectiousness of the virus may have contributed to a preference for greater distance from others during the pandemic. This link replicates the findings of Iachini et al., who found an association between DD boundaries and perceived, but not actual, COVID-19 infection risk during the early phases of the pandemic [14], and is in line with finding from Serino and colleagues, who found that germ aversion was predictive of the segregation between near and far space [44]. Findings from a recent cross-cultural study by Croy et al. also show that perceived vulnerability to disease predicts larger boundaries of DD regardless of the interaction partner [45]. This suggested association between perceived threat from COVID-19 and DD boundaries might be mediated by social distancing. A study by Holt et al. similarly found that the boundary of DD was correlated with social distancing behaviour (ratings of “I stay at least 6 feet away from people when I am outside”) [5]. In line with this, data have shown that social distancing measures has led to the segregation of peripersonal space between individuals which they describe as an implicit form of freezing behaviour in social contexts [46].

Unlike previous attempts looking at DD, which make use of visual cues to determine preferences for the space between ourselves and others, our approach takes advantage of the use of triggers in the auditory domain to manipulate perceived threat. Specifically, we measured DD when no visual information about the approaching person was available to the participants and used an auditory task where participants heard footsteps of a person approaching them. Building on work by de Vignemont and Ianetti who describe peripersonal space as serving two functions [4], this task measures both, with the former operationalized as reachable space, and latter as interpersonal comfort space. This modified version of the classical visual paradigms is effective in measuring DD, and has the benefit of eliminating any interactions and confounds with the idiosyncrasy of the approaching person [43]. Neuhoff (2018) explains that while the visual system provides more accurate and precise estimates of arrival for the approaching threat, the auditory system functions as an advanced warning system that facilitates the decision about whether to direct the eyes toward the looming object, or whether immediate evasive actions are needed [47]. Unlike vision, the auditory system functions well when visibility is poor and when objects are occluded or are out of the line of sight. This is especially pertinent within the context of a pandemic, where there is no visual information as to where and from whom disease transmission might be happening (e.g. invisible respiratory droplets). By allowing us to represent our bodies as fundamentally in relation to something else, audition grants us the ability to flexibly, and with high-resolution, combine and segregate events from the sounds produced by others or external events [48]. Successful interaction with looming objects entails a multisensory integration [47,49], and needs to be further deciphered in future studies looking at DD preferences, specifically as they relate to tactile events in the post-COVID-era.

### 3.2 Perceived threat predicts both touch avoidance and approach behaviours

Based on a subset of items from the Touch Experiences and Attitudes Questionnaire [50], we probed general attitudes towards touch within the context of the COVID pandemic. Our data shows that the greater the perceived threat from COVID-19, the higher the fear of interpersonal touch (FIT). Investigating differences across our two cohorts, we see that while within the UK sample perceived threat is associated with higher aversion to interpersonal touch, for the German sample the effect was reversed. The data from the UK was collected between 02.12.20 and 13.01.21 which coincided with the first weeks of the COVID-19 vaccine rollout in the UK, as well as the emergence and rapid spread of the Alpha variant (B.1.1.7) of the SARS-CoV-2. With the data from Germany, however, data collection started slightly later and was collected across a longer period (7.01.21–19.03.21) during which Germany experienced a new set of lockdown measures, which were gradually lifted. Given the dynamic nature of the pandemic, information regarding the virus and its effects as well as governmental intervention, cross-country differences are to be expected with our data corroborating reports of increased concern over the risk of COVID-19 in the UK [51] and as such predictive of greater fear of interpersonal touch.

Beyond the relationship between perceived threat and general touch avoidance, our data further shows that perceived threat from COVID-19 also predicts increased perceived pleasantness, likeability, and ratings of comfort for “safe” household touch (but not outsider touch). This approach-like behaviour is in line with the findings of Meijer and colleagues who showed that the severity of COVID-19 regulations predicted perceived pleasantness of observed touch [52]. Meijer et al. note that longing for touch is predictive of increased perceived pleasantness of both CT-optimal and CT-suboptimal stroking speed. Similarly, von Mohr et al. found that the more days practising COVID-19-related social distancing, the more individuals wanted to experience touch [21]. As such it could be that heightened feelings of perceived threat from COVID-19 led to greater hunger for touch, resulting in a higher appraisal of it. Interestingly, perceived threat was not associated with reduced pleasantness of outsider touch, perhaps because touch from people outside of the household was not appealing to anyone, regardless of perceived threat levels in the COVID-19 context, which would be in line with Croy et al., who report that perceived vulnerability to disease is linked to lower touch diversity [45]. This explanation, however, contrasts with the finding of von Mohr where they compared intimate, friendly, and professional touch and show that under COVID-19 regulations people also craved friendly and professional touch, highlighting the importance of variety in touch interactions [21]. In the current study, we probe only two classes of touch, safe and threatening, as a function of who is touching us and where [53]. Participants perhaps considered only the two above-mentioned categories regardless of their more nuanced relationships. This explanation is consistent with data reported by Meijer and colleagues who did not observe a difference in longing for touch levels between participants who had a bad relationship with their housemates and those who had a good relationship [52]. How touch was perceived across household or stranger touch was probed across three different dimensions of touch perception, namely comfort, likability, and pleasantness. Our results show that all three scales are modified by threat perception. Owing to the wide range of studies that have been published on the impact of the pandemic on our touch preferences and perception[21,[52], [53], [54]] [45] [21,[52], [53], [54]], it may be necessary to reevaluate the kinds of scales we use to capture our feelings about touch. Perhaps there is a binary distinction in touch perception within the context of a pandemic: it is either acceptable or not, as a function of perceived threat, and if it passes the acceptability threshold, then touch is perceived to be pleasant, likeable, and comfortable. Such an explanation would be in line with Cartaud et al. who found that threatening emotions were perceived as such, only if they passed the border of comfort space [55].

The present study employed videos of vicarious touch that have been used to differentiate behaviourally and neurally between observed CT-optimal and suboptimal stroking speeds [56,57]. Based on findings that perceived pleasantness of vicarious touch interacts with a stroking velocity akin to gentle stroking [58], we hypothesised that the pleasantness ratings of CT-optimal touch would be greater than that of CT-non-optimal touch. Our findings, however, suggest no difference between the pleasantness, likeability, or comfort ratings for CT-optimal and non-optimal touch. Similar to these dimensions by which we experience touch, we propose that all touch (physical contact) is threatening within the context of a pandemic, regardless of its velocity. This hypersensitivity is reminiscent of previous findings by Morrison that CT-afferent activation blunts the impact of repeated stressors and could thus be a rewarding signal of safety [59].

### 3.3 Perceived but not real threat influence both discomfort distance and touch behaviours

Probing the nature of threat but also individual differences in protective behaviours, our design distinguished between the influence of perceived versus real threat from COVID-19 on interpersonal boundaries. Our data shows that overall increased *perceived* threat predicted larger boundaries of DD, with that this effect is more pronounced in the UK sample compared to German. We see a similar overall effect on reaching distance, a space defining goal directed action. Again, perhaps related to the difference in context across the two countries, the effect is starkest in the UK sample with little change in reaching distance as a function of perceived threat in German respondents. As such, beyond the observed individual plasticity to interpersonal boundaries as a function of context, there also seems to be social moulding of that space such that different countries behave differently. While Sorokowska et al. reported no effect on DD of country-level parasitic stress [20], given the relation between disease-avoidance mechanisms and social cognition [60], it would be particularly interesting to investigate cross-sectional DD preferences from a cultural perspective, taking into account the dynamicity of the COVID-19 pandemic.

By contrast, *real* threat posed by COVID-19 (operationalized by age) was associated with smaller boundaries of DD and RD. In line with previous findings, despite being objectively at greater risk, older individuals have been reported to engage in fewer protective behaviours [61]. The underlying reason might be that, as several studies note, older people experienced more loneliness during the pandemic (e.g. Ref. [62]). This result also resonates with Iachini and colleagues, who reported that DD expansion was predicted by perceived but not actual threat [14]. As the present study is not capturing the potential mediators of the effect of age on DD, such as mental health and loneliness, future studies should disentangle whether indeed age reflects the real threat from COVID-19, especially given that our proxy for interpersonal boundaries was response time, which in older individuals has been shown to be slower [63]. Interestingly, age was associated neither with fear of interpersonal touch nor with pleasantness of touch. This may simply be an issue of power due to the relationship between the sample's age spread and the tools used to capture changes in touch attitudes. In the present study we restricted our stimuli to those representing either safe “household” or threatening “outsider” touch. Future research into age related differences in touch avoidance and approach behaviours should aim to further detail how this varies as a function of types of touch. Similarly, the use of the Fear of Interpersonal Touch questionnaire items produces a fairly binary view of either wanting or not wanting touch, but here again there might be a nuance as to what kind of touch individuals were either averse to or not. Together this opens the door to further investigation both at the individual level but also a function of social norms (across countries or cultures).

### 3.4 How perceived threat changes the space around us and how we interact through touch

Defence of the body surface entails a collection of processes bound together by similar sensorimotor computations and the goal of protecting the body from attack or collision [64]. Indeed, the safety margin surrounding the body within which threatening stimuli trigger efficient actions aimed at self-protection is termed defensive peripersonal space (PPS) [19]. Accordingly, if a stimulus that might pose a threat to the body enters the PPS, defence mechanisms are activated, highlighting the role of PPS in regulating social spaces [3]. Resonating with the homeostatic theory of social interaction that the sensorimotor characteristics of the PPS provides a spatial reference for the maintenance of proper distancing [2], this begs the questions of how the defensive spaces adapt to novel threats through these interactions, and whether, in turn, this influences the way those interactions are perceived. Welsch and colleagues report that post-pandemic DD remained enlarged [9], which suggests that while people can rapidly adapt to new DD norms, enlarged preferences may linger, at least in part, even after restrictions are lifted. Similarly, while some studies argue for a hunger for touch as a function of social restrictions imposed by COVID-19 (e.g. Ref. [54], others claim that such constraints have altered the way people interpret touch, transforming it into a perceived threat signal that could extend to diminished positive responses to observed instances of touch [65]. In this context, our data highlight the marked difference in how threatening “outsider” and safe “household” touch is evaluated. We posit that adaptations in our defensive peripersonal space during the COVID-19 pandemic and the changes in our preferred boundaries of DD mediate the relationship between perceived threat from the virus and perceived pleasantness of vicarious affective touch. Although our mediation analyses did not show significant results, future studies should investigate this posited relationship with a larger sample size while taking into account both contextual and individual level differences. Moreover, the plasticity and duration of these changes remains to be investigated.

Exploring the relationship between threat and interpersonal boundaries, studies investigating threat proximity with looming stimuli have revealed a correlation between preferred DD and BOLD correlation strength in regions related to personal space regulation and defensive actions [66]. These results can be taken to suggest that the interaction of PPS and defensive PPS might underlie the regulation of DD and that personal space intrusions fundamentally involve defensive reactions. Candidate brain regions that allow for these plastic changes to the space we maintain between ourselves and others and interpersonal behaviours such as touch may include the amygdala [38]. Indeed, lesion studies suggest that the amygdala allows for the detection of socially and emotionally salient aspects related to physical distance between ourselves and others. Separately but relatedly, touch research has suggested that a key role played by the CT afferent system, so intertwined with social affective touch, is for interoception [67] and therefore potentially protection. Along with the other evolutionarily ancient unmyelinated fibres for itch and pain (along with olfaction), CT mediated touch may govern both approach and avoidance behaviour. Based on the social salience hypothesis [32], and recent reports of an anticipatory autonomic response [68], CT affective touch is expected to enhance susceptibility for social stimuli by modulating central network connectivity [69,70]. Connecting these parallel lines of investigation, it would therefore be interesting to investigate the effects observed in the present study in the context of tactile defensiveness [37], especially given the reported involvement of brain areas including the amygdala in the processing of social threat perception [71], interpersonal space [72] and affective touch [73].

## 4 Conclusion

Our results demonstrate that greater perceived threat from COVID-19 is associated with larger boundaries of preferred interpersonal space. We also found a positive association between perceived threat and pleasantness and liking of, and comfort with household touch, but no association with outsider touch. These results suggest acceptability as a defining characteristic of how touch is perceived, thus marking household touch to be safe, and outsider touch to be threatening, regardless of stroking speed. We finally discuss our results within the context of the spaces surrounding our bodies and posit a potential mechanism whereby the relationship between threat perception and perceived pleasantness of affective touch is mediated by the changes in preferred boundaries of interpersonal space and by the adaptations in our defensive peripersonal space. Rather than focusing on the purely “positive” and prosocial functions of affective touch, this study bolsters a novel perspective that this same system may be at work when socially-relevant information cues danger rather than trust, shifting the balance from sensory seeking to sensory avoidance.

## 5 Limitations of the study

The findings of this study should be considered in light of their limitations. The pandemic regulations mandated that this study be conducted online, which biased our sample to younger adults. Indeed, almost half of our study population consists of people younger than twenty-five, who, combined with the fact that they were less exposed to inevitable social contacts, were at lower risk of infection. This might have underestimated the DD changes associated with actual threat, as marked by age. Moreover, this study does not report pre-pandemic baselines for attitudes towards social touch and therefore cannot offer conclusions regarding the precise relationship between threat from COVID-19 and social touch perception. As we are now resuming our social activities similar to pre-pandemic norms, future research should compare these findings with DD preferences focusing more generally on perception of other kinds of threat. We also acknowledge that our questionnaire for Fear of Interpersonal Touch is not validated but rather tailored to the specific context of the pandemic. We approached the Touch Experiences and Attitudes Questionnaire from which we obtained our questions as a tool that is being adapted as a function of our increasing understanding of the complexities of attitudes towards touch but also to the needs of specific research questions. We therefore selected the items that were most relevant. Finally, due to the fast-evolving nature of the pandemic and the resulting time limitations, this study was conducted only on samples from two countries. As such, investigating the social and cultural moulding of sensory avoidance and seeking behaviours within the context of threat would require a larger participant pool across more countries.

## 6 Inclusion and diversity

We support inclusive, diverse, and equitable conduct of research.

## 7 STAR methods

### 7.1 Resource availability

#### 7.1.1 Lead contact

Further information and requests for resources and reagents should be directed to and will be fulfilled by the lead contact, Merle Fairhurst (merle.fairhurst@gmail.com).

#### 7.1.2 Materials availability

This study did not generate new materials.

#### 7.1.3 Data and code availability

All questionnaire and subjective report data can be found on Mendeley Data (Abra, Yasemin; Mirams, Laura; Fairhurst, Merle (2024), “The space between us: the effect of perceived threat on discomfort distance and perceived pleasantness of interpersonal vicarious touch”, Mendeley Data, V3, https://doi.org/10.17632/m62td4fcxz.3) and are publicly available as of the date of publication. This paper does not report original code. Any additional information required to reanalyse the data reported in this paper is available from the lead contact upon request.

### 7.2 Study participants details

A total of 96 participants were included in the study (44 male, 52 female; M_age_ = 34.9, max_age_ = 74, min_age_ = 19). They were recruited from the UK (n = 40) and Germany (n = 56). A priori power analysis was conducted using G*Power 3.1.9.7 to determine the necessary sample size for detecting a medium effect size (f^2^ = .15) in a multiple linear regression analysis with 6 predictors. The analysis aimed to achieve a statistical power of .80, with an alpha level set at .05. The results indicated that a total sample size of 98 participants would be required to achieve these parameters. 4 participants were excluded from the 100 who completed the study due to reporting countries of residence outside of the UK and Germany. Hence we find using a post hoc power analysis that a total sample size of 96 yields an achieved power of .79.

An additional 73 participants were automatically rejected from the study due to reaching the set time limit of 1 h and 15 min and 1 participant due to being underage. The study was created and carried out using the online platform Gorilla Experiment Builder [74]. Collected sensitive data via the Gorilla.sc site is anonymous and protected from third parties. Participants took part in the study via a link, whereupon they were first informed about the use of their data and the approximate duration of the study (15 min) and asked for their consent. Participants were required to be aged 18 and over. The study was carried out on mobile and stationary devices with IOS, Android, Windows, or Linux operating systems. The data from the UK and Germany were collected between 02.12.20 and 13.01.21 and 07.01.21–19.03.21, respectively. The study was approved by the Ethics Commission of the Universität der Bundeswehr München.

### 7.3 Method details

#### 7.3.1 Questionnaires

To measure fear of interpersonal touch, nine items from the Touch Experiences and Attitudes Questionnaire [50] were amended to assess whether participants feel comfortable or uncomfortable about interpersonal touch with friends and family not from their household and strangers (Table S1). As the original questionnaire has 57 items, we selected the items that were most relevant to our study aims in order to reduce the burden on participants, and then adapted these to better fit the context of COVID-19. The participants' total score was taken. To ascertain actual risk to COVID-19, participants were also asked for demographic information (age, gender, occupation, current country of residence). Participants were asked to complete the Perceived Coronavirus Threat (PCT) Questionnaire [75]. This measure includes 6 items, assessing perceived threat from Coronavirus (e.g.,“Thinking about the Coronavirus makes me feel threatened”, and “I am stressed around other people because I worry I will catch the Coronavirus”). The participants’ total score was taken. Content was translated and then back translated by two native speakers per language and compared to check for cross-language equivalence.

#### 7.3.2 Interpersonal boundary task

Participants listened to six audio clips consisting of the sound of footsteps approaching them from the front [43]. The walking sound effects were generated and recorded using Unity 3D (Unity Technologies, 2020). Free footstep sounds were downloaded from https://freesound.org/and were split into single files, one file per footstep. A unity script loaded the footstep audio files when a walking animation was executed and synchronised each sound with the animation. A unity scene with an audio reverb zone was created with a maximum distance of 36 m. An audio listener was located inside the reverb zone which allowed one to gradually change the footstep sound effect as the animation slowly moved closer to the target. The output was generated using the Unity Recorder tool.

In half of the trials, participants were asked to indicate “Stop” when the person heard approaching made them feel uncomfortable with the distance between themselves and the approaching person. In the other half of the trials, participants were asked to indicate “Stop” when they felt like they could touch the person they heard coming towards them. The order of these two tasks was counterbalanced across participants. In each case, the response times (RT) in milliseconds, i.e. the time taken to indicate “Stop”, were measured, with a longer RT a proxy for smaller boundaries of space (see Fig. 1). The task distinguishes between types of space representations: the former representation being involved in protecting the body, and the latter in goal-directed action. As such, we dissociate between discomfort and reaching distances. Here we adapted the auditory task by Vagnoni and colleagues to capture RD to compare with DD [43,76].

#### 7.3.3 Perception of vicarious affective touch

Participants were presented with four video clips of a person's forearm being stroked by another person's hand. In the videos presented, the gender of the person being touched was matched according to the self-declared gender of the participant; in all cases the person performing the stroking was female. The speed of the stroking movement was either at the OPTIMAL for activation of C-tactile afferent fibres (i.e. 3 m/s) or significantly faster at a C-tactile afferent SUBOPTIMAL stroking speed (i.e. 30 m/s [56]). In two of the video clips, participants were told to imagine two different contexts: being touched like that *by a friend or family member from their household* (HOUSEHOLD). In the other two trials, participants were asked to imagine being touched like that *by a friend or family member who is outside of their household* (OUTSIDER)*.* By comparing household familiar touch with outsider familiar touch, we control for familiarity while manipulating the level of possible uncertainty as to “safety”/contagion. After each video, they were told to rate the experience using three visual analogue scales: 1) How did that video clip make you feel (anchored, “very uncomfortable” to “very comfortable”); 2) How do you think the person being touched would rate the touch? (anchored, “very unpleasant” to “very pleasant”), 3) How much would you like to be touched like that? (anchored, “not at all” to “a lot”).

#### 7.3.4 Procedure

Following the demographic questionnaire, only the participants who were over the age of 18 were allowed to continue the study. Participants then completed the interpersonal boundary task and the ratings of vicarious touch with the order of these tasks counterbalanced between participants. Participants then completed a second set of questionnaires including Fear of Interpersonal Touch Questionnaire and Perceived Coronavirus Threat Questionnaire.

#### 7.3.5 Data handling and analysis

Data were extracted from the Gorilla.sc platform, organised and analysed using R Statistical Software (v4.1.0; R Core Team, 2021) [77]. After performing model diagnostics (linearity, residual normality, and homoscedasticity) and transforming the measures where necessary, linear regressions, linear mixed effect models and mediation analyses were employed in order to ascertain the associations between perceived and actual threat from COVID-19, DD, RD, and ratings of vicarious touch (see Table S2 for descriptives).

## CRediT authorship contribution statement

**Yasemin Abra:** Writing – review & editing, Writing – original draft, Visualization, Formal analysis, Data curation. **Laura Mirams:** Writing – original draft, Formal analysis, Data curation, Conceptualization. **Merle T. Fairhurst:** Writing – review & editing, Writing – original draft, Visualization, Supervision, Funding acquisition, Formal analysis, Data curation, Conceptualization.

## Declaration of competing interest

The authors declare the following financial interests/personal relationships which may be considered as potential competing interests:Merle Fairhurst reports financial support was provided by 10.13039/501100001659Deutsche Forschungsgemeinschaft (10.13039/501100001659DFG). Merle Fairhurst reports financial support was provided by 10.13039/501100002347Federal Ministry of Education and Research of Germany. If there are other authors, they declare that they have no known competing financial interests or personal relationships that could have appeared to influence the work reported in this paper.
