# Supplementary material for: The space between us: The effect of perceived threat on discomfort distance and perceived pleasantness of interpersonal vicarious touch
Source: Heliyon. 2024 Aug 16;10(16):e36487. doi: 10.1016/j.heliyon.2024.e36487 (PMC11388568; doi:10.1016/j.heliyon.2024.e36487)
Supplement: Multimedia component 1 [file mmc1.docx]

**SUPPLEMENTARY MATERIALS**

**The space between us: the effect of perceived threat on discomfort distance and perceived pleasantness of interpersonal vicarious touch**

^1^Yasemin Abra

^2^Laura Mirams

^1^Merle T. Fairhurst*

1. Centre for Tactile Internet with Human-in-the-Loop (CeTI), 6G life, Faculty of Electrical and Computer Engineering, Technische Universität Dresden,
2. School of Natural Sciences and Psychology, Liverpool John Moores University, Liverpool, United Kingdom

**Corresponding author**

[merle.fairhurst@gmail.com](mailto:merle.fairhurst@gmail.com)

**Table S1: Fear of Interpersonal Touch Questionnaire**, amended from the Touch Experiences and Attitudes Questionnaire (Trotter et al., 2018)

| **Items** |
| --- |
| I feel wary of hugging friends and family when I am saying goodbye. |
| I would feel uncomfortable if a friend or family member greeted me with a kiss on the cheek. |
| I would feel comfortable linking arms with a friend or family member if we were on a walk together. |
| If someone I didn’t know very well put a friendly hand on my arm, it would make me feel uncomfortable. |
| I am not worried about giving my friends and family a hug when we meet up. |
| I try to avoid making physical contact with my friends and family when I am with them. |
| It would not bother me if a close friend put their arm around me on a walk. |
| Before the COVID-19 pandemic, I liked it when my friends and family greeted me by giving me a hug. |
| Before the COVID-19 pandemic, I usually hugged my family and friends when I was saying goodbye. |

**Table S2: Descriptives of Variables**

**
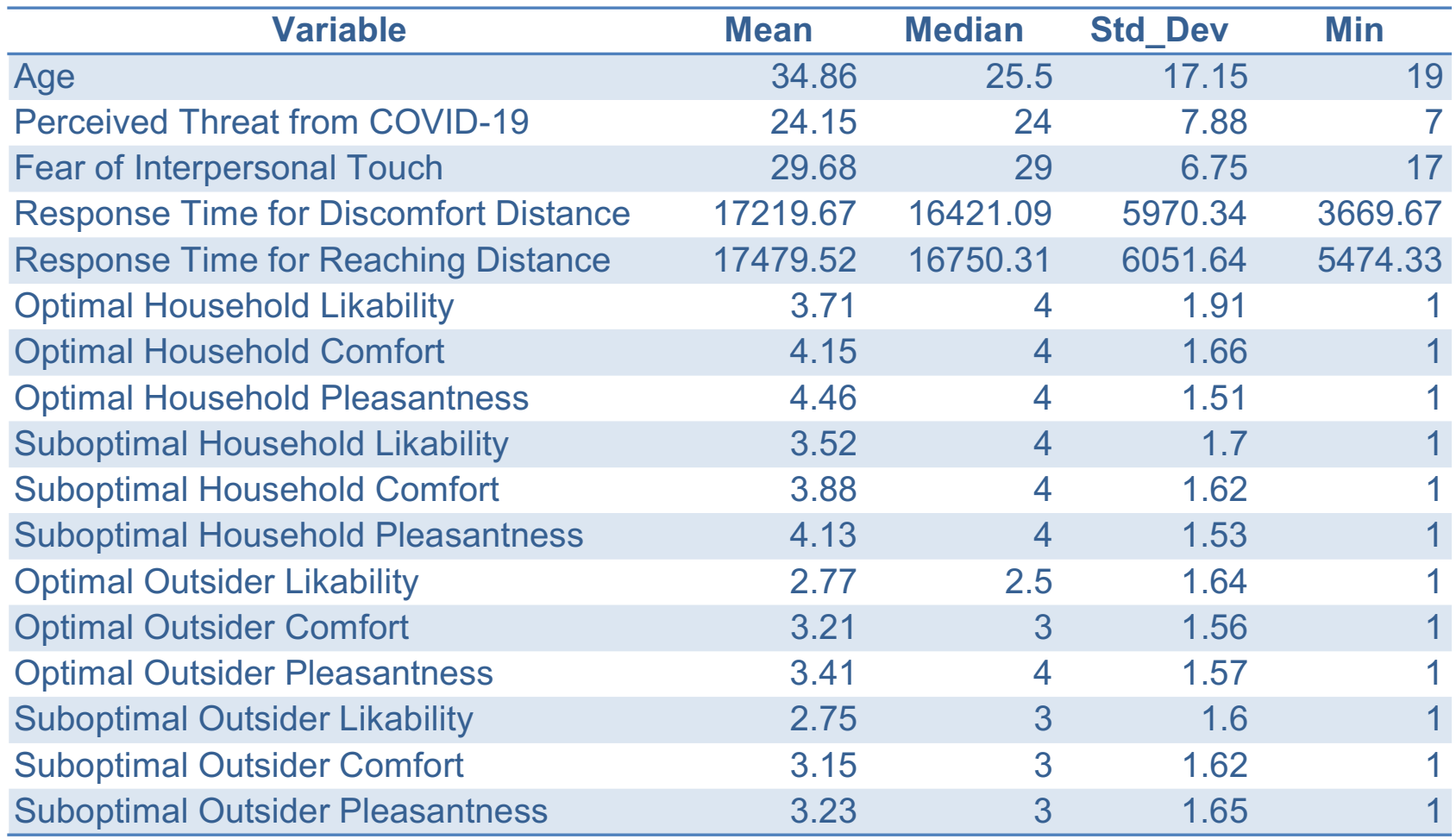
**

**Figure S1:** **The influence of perceived threat reaching distance as a function of country of residence.** For visual purposes, response times in seconds are reversed to indicate reaching distance. Plot includes regression lines with shaded 95% confidence intervals.

**
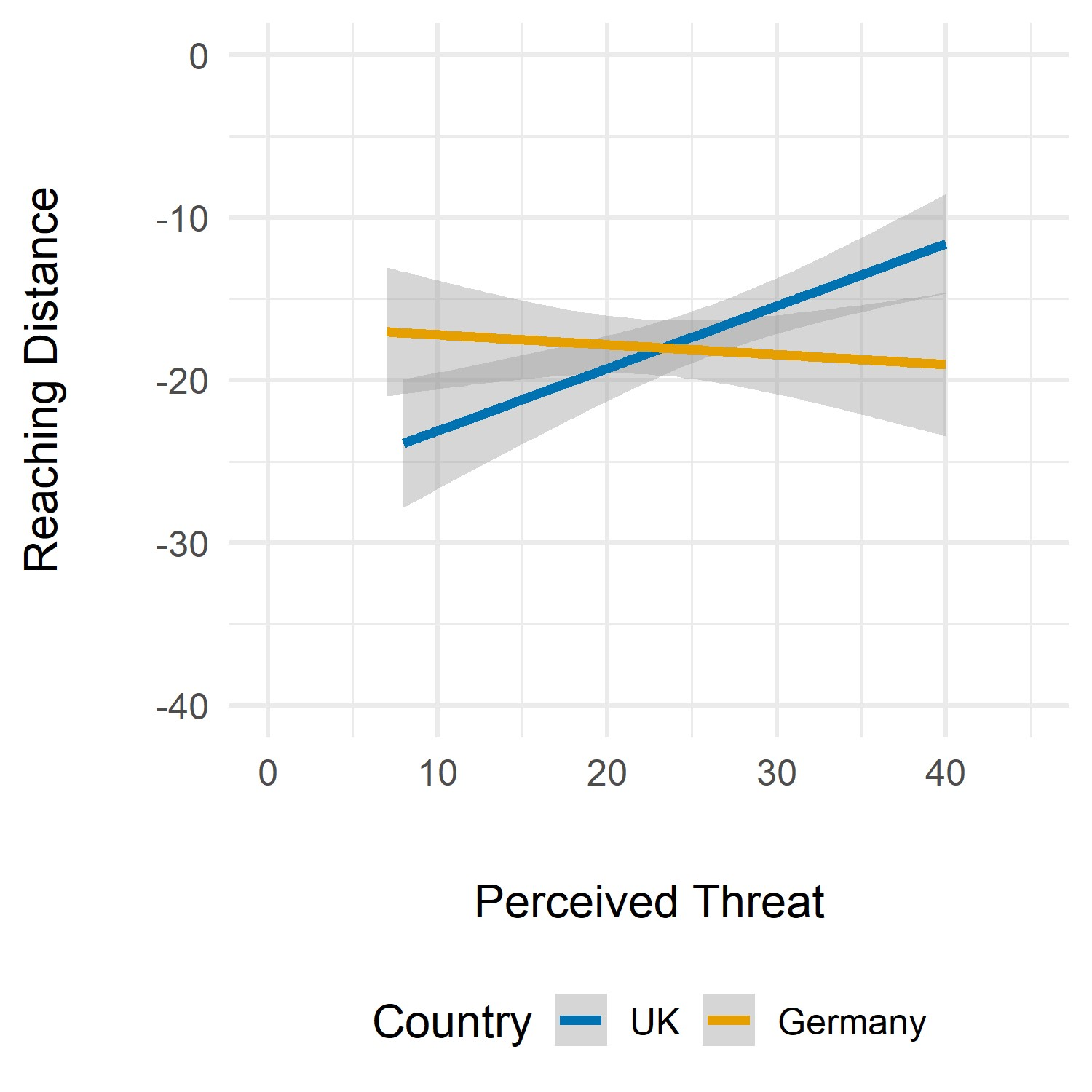
**

**Figure S2: Vicarious touch ratings as a function of perceived threat from COVID-19, differentiated by Context (Household and Outsider) and Stroking Speed (CT-optimal and CT-suboptimal).** Plots include regression lines.

####

#### **
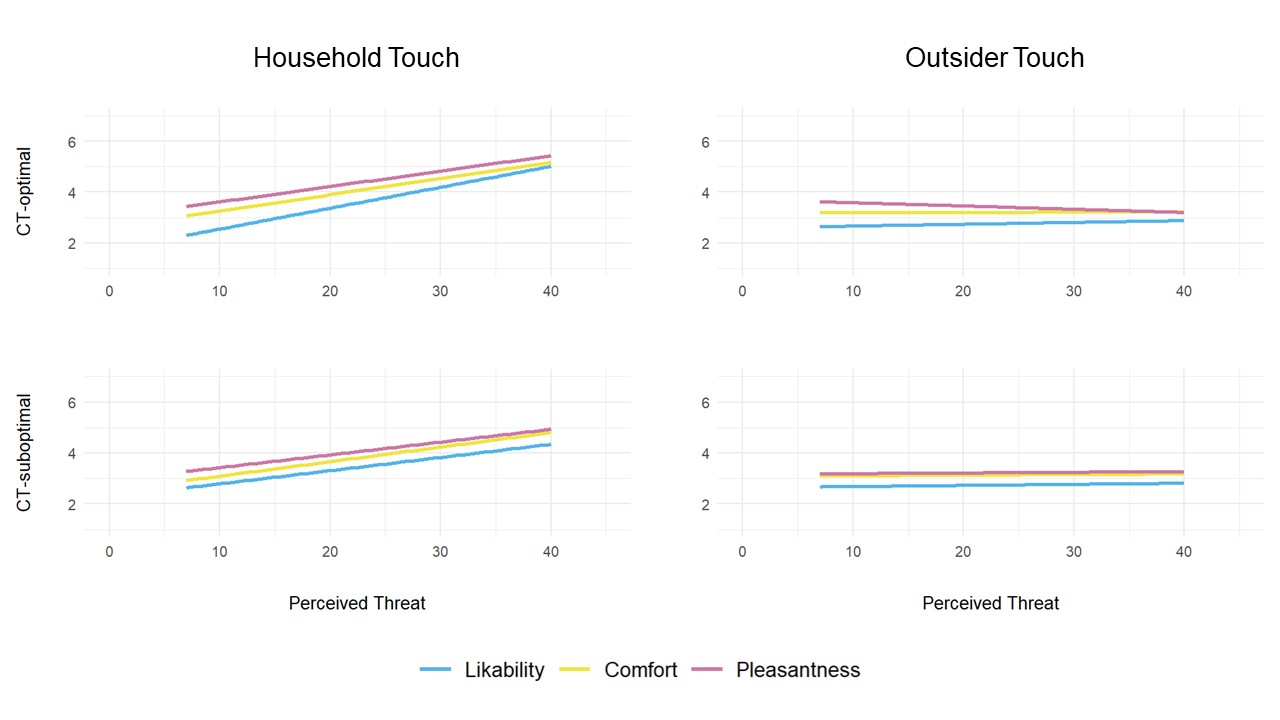
**
